# The National Burden of Influenza‐Associated Respiratory Illness Across Levels of Severity in Egypt, 2016–2019

**DOI:** 10.1111/irv.70061

**Published:** 2024-12-25

**Authors:** Manal Fahim, Ola Deghedy, Walaa Alim, Reham Kamel, Hossam Hassan, Amira Mohsen, Rania Attia, Hala Abou El Naja, Salma Afifi, Stefano Tempia, Amr Kandeel

**Affiliations:** ^1^ Preventive Sector Ministry of Health and Population Cairo Egypt; ^2^ Egypt Country Office World Health Organization Cairo Egypt; ^3^ Eastern Mediterranean Regional Office World Health Organization Cairo Egypt; ^4^ Ministry of Health and Population Cairo Egypt; ^5^ Global Influenza Program World Health Organization Geneva Switzerland

**Keywords:** burden, influenza virus, severe acute respiratory infection, surveillance

## Abstract

**Introduction:**

Influenza burden (IB) estimates are crucial for monitoring disease trends, allocating limited resources, and promoting influenza vaccination. However, IB in Egypt is poorly understood. This study estimates the mean‐seasonal IB in Egypt, across levels of severity by age and regions using sentinel surveillance data between 2016 and 2019.

**Methods:**

Influenza surveillance was implemented among patients hospitalized with severe acute respiratory infection (SARI) at eight sentinel hospitals across Egypt. We estimated the influenza‐associated SARI hospitalization in two governorates and then extrapolated nationally using the World Health Organization (WHO)–recommended methods. Thereafter, we estimated IB‐associated mild/moderate illness and deaths using IB pyramid tool developed by WHO and the John Hopkins Center for Health Security. Rates were reported per 100,000 population.

**Results:**

The estimated mean seasonal number of influenza‐associated mild/moderate illness, hospitalized noncritically and critically ill patients, and deaths was 16,425,938 (95% CI: 1,150,888–40,409,614), 30,335 (95% CI: 9971–670,288), 9110 (95% CI: 580–16,321), and 2660 (95% CI: 154–9908), respectively. The highest rate of influenza‐associated mild/moderate illness was among aged 5–14 year (22,932; 95% CI: 825–25,546.3), whereas the highest rates of severe influenza were among aged > 65 years (hospitalizations: 159.4, 95% CI: 121.7–205.0; deaths: 56.0, 95% CI: 0.6–111.0). Children aged < 5 years also experience high rates of influenza‐associated hospitalization (52.0, 95% CI: 43.0–62.0).

**Conclusions:**

The WHO method estimated a high burden of severe influenza among aged ≥ 65 and aged < 5 years in Egypt. To reduce severe IB, increased influenza vaccine uptake together with enhanced immunization strategies implementations among the elderly and children are warranted.

## 1 Introduction

Influenza remains a high public health priority because of its potential to cause pandemics [1]. In addition, seasonal influenza viruses cause 3.5 million cases of severe illness and between 290,000 and 650,000 deaths worldwide each year [2]. A higher risk of severe influenza is experienced by young children, the elderly, pregnant women, and people with underlying health conditions [2, 3, 4].

Estimates of the influenza disease burden are critical to foster influenza vaccination, especially among high‐risk groups. Such estimates are particularly important in low‐ and middle‐income countries to guide the allocation of limited resources. Nonetheless, several challenges exist when estimating the influenza disease burden, including the difficulty of distinguishing influenza from other respiratory illnesses without comprehensive laboratory testing and the inability of surveillance to capture the whole spectrum of illnesses associated with influenza virus infection [5].

Egypt started monitoring influenza virus circulation in 1999 through sentinel surveillance among outpatients with influenza‐like illness (ILI). In 2007, sentinel surveillance was expanded to include in‐patients with severe acute respiratory infection (SARI). In 2009, acute respiratory infections (ARIs) were added to the notifiable diseases list of the National Electronic Disease Surveillance System (NEDSS) [6]. In Egypt, influenza virus circulation is observed throughout the year with heightened activity from October to March and a decrease in activity during the summer period between June and August [6].

The incidence of influenza‐associated SARI hospitalization has been estimated in Damanhur district in Lower Egypt in 2013 [7]. Nevertheless, estimates of the national burden of influenza‐associated illness in Egypt are lacking, as are estimates based on severity levels. In this study, we sought to estimate the mean‐seasonal influenza respiratory disease burden across levels of severity (i.e., mild/moderate illness, hospitalization, and deaths) by selected age strata and regions of Egypt during 2016–2019.

## 2 Methods

### 2.1 Study Settings

Egypt is a lower middle‐income country situated in North‐East Africa and characterized by a dry and hot climate. Egypt is administratively divided into 27 governorates, of which four are predominately urban (Alexandria, Port Said, Cairo, and Suez) and 23 have both urban and rural inhabited areas. The latter governorates are located in three regions: Lower Egypt, which includes nine governorates (Beheira, Dakahlia, Damietta, Gharbia, Ismailia, Kafr el‐Sheikh, Menofia, Qualyubia, and Sharqia); Upper Egypt, which includes nine governorates (Aswan, Assuit, Beni Suef, Fayoum, Giza, Luxor, Minya, Qena, and Sohag); and five Frontier governorates (Matrouh, New Valley, North Sinai, Red Sea, and South Sinai) are located along the Egyptian border [8]. The Egyptian healthcare system comprises a variety of public and private healthcare providers. The government ensures basic universal healthcare coverage, although private services are also available for those with the ability to pay.

### 2.2 Overview of the Estimation Approach and Data Sources

We estimated the average number and rates of influenza‐associated respiratory illness in Egypt over three influenza seasons (September 2016–August 2019; September through August mostly representing the influenza season in Egypt) across three levels of severity, namely, mild/moderate illness, hospitalization, and deaths. Average rates and numbers were estimated overall and within the following age strata: < 5, 5–14, 15–49, 50–64, ≥ 65, and ≥ 5 years of age. Rates were reported per 100,000 population. We selected the 2016–2019 influenza seasons based on availability of robust and systematic sentinel surveillance data from eight sentinel hospitals situated across Egypt. Robust data on ARI hospitalization from all public hospitals in the country were available from the NEDSS during the same period. For the estimation of burden of influenza‐associated illness, we excluded the coronavirus disease 2019 (COVID‐19) pandemic years (2020–2022) because during that period, influenza virus showed abnormally low circulation pattern (results not shown). In addition, influenza sentinel surveillance implementation was suboptimal during the COVID‐19 pandemic.

First, we estimated the rate of SARI hospitalization at selected SARI sentinel hospitals situated in Damietta Governorate (in Lower Egypt) and Aswan Governorate (in Upper Egypt) using the World Health Organization (WHO) manual for estimating the burden of seasonal influenza [9]. Second, we extrapolated the SARI hospitalization rates obtained in the Damietta and Aswan Governorates to national level and estimated the influenza‐associated SARI hospitalization rates and numbers using a previously described methodology that leverages the differential prevalence of known risk factors for pneumonia and healthcare seeking behavior between Governorates [10]. Last, we used the influenza pyramid tool developed by WHO and the John Hopkins Center for Health Security to obtain estimates of influenza‐associated mild/moderate illness and deaths [11].

The data sources used to estimate the national rates and number of influenza‐associated SARI hospitalizations are provided below:

- *NEDSS*: We extracted data on patients admitted with ARI at all public hospitals in the Damietta and Aswan Governorates during the study period (September 2016–August 2019). This period spans three influenza seasons in Egypt, where the virus circulates year‐round with peaks from October to March and low‐level circulation between June and August.
- *SARI sentinel surveillance*: We conducted influenza sentinel surveillance among inpatients with SARI at eight sentinel hospitals during the study period, namely, Abbasia chest and Abbasia infectious diseases hospitals in Cairo governorate, Alexandria infectious diseases hospital in Alexandria governorate, Aswan infectious diseases hospital (AIH) in Aswan governorate, Damietta chest hospital (DCH) in Damietta governorate, Mahalla infectious diseases hospital in Gharbia governorate, Menia infectious diseases hospital in Menia governorate, and Shebin infectious diseases hospital in Menofia governorate. A case of SARI was defined as a patient having a history of fever or measured fever of ≥ 38°C and cough with onset within the last 10 days and requiring hospitalization [12]. The procedures of this surveillance program have been previously described [13]. Briefly, trained surveillance nurses completed case report forms that included demographic, clinical, and epidemiological information for all enrolled SARI cases. In addition, respiratory specimens (nasopharyngeal and oropharyngeal swabs) were collected from all enrolled patients, placed in the same vial containing universal transport medium, stored at 4°C–8°C, and transported to the WHO‐recognized National Influenza Center (MoHP Central Public Health Laboratory) or the regional laboratories within 72 h of collection for testing. Specimens were tested for influenza A and B viruses using a real‐time reverse transcriptase polymerase chain reaction assay [14]. Influenza A‐positive samples were further subtyped [13]
- *Prevalence of risk factors for pneumonia and healthcare seeking behavior*: We obtained the governorate‐specific prevalence of known risk factors for pneumonia such as indoor smoking, air pollution, low birth weight, malnutrition, and nonexclusive breast feeding, as well as data on healthcare seeking behavior for sexually transmitted diseases by Governorate from the 2015 Egyptian Demographic and Health Survey (DHS) [8].

#### 2.2.1 Population Denominators

We obtained age‐ and year‐specific midyear population denominators by governorate from projections of the 2017–2019 Egyptian census obtained through the Ministry of Health and Population Information Center. Egypt had a population of 96,592,371 individuals, of which 23,516,826 (24.7%) were children aged < 5 years [15].

### 2.3 Estimation of National Number and Rate of SARI and Influenza‐Associated SARI Hospitalizations

#### 2.3.1 Step 1: Estimation of SARI Hospitalization Rates in Damietta and Aswan Governorates

Damietta and Aswan governorates (considered the base governorates in our estimation approach) were selected for the estimation of SARI hospitalization rates because the majority (> 90%) of hospitalized SARI patients in these governorates seek care in public hospitals, which are included in the national routine surveillance system (NEDSS). First, using the NEDSS data, we estimated the source population of the sentinel hospitals (DCH and Aswan infectious diseases hospital) located in each governorate by dividing the number of SARI hospitalizations at each sentinel hospitals by the total number of SARI hospitalizations admitted to all governmental hospitals located in the base governorates. Thereafter, we multiplied this proportion by the midyear population estimate of each governorate. Lastly, we calculated the SARI hospitalization rate for the sentinel hospitals, by dividing the total number of SARI cases in the sentinel hospital by the hospital source population obtained as described above. We used the SARI hospitalization rates at the two sentinel hospitals as a proxy for the respective governorates as previously described [9]. The equation used for the calculation of the SARI hospitalization rates for the sentinel hospitals located in the base governorates is provided below:

$${\mathrm{RS}}_{H,B}=\frac{{\text{SARI}}_{H,B}}{{\mathrm{Pop}}_{B}},$$

where 𝑅𝑆𝐻,𝐵 = number of total cases meeting SARI case definition hospitalized at the sentinel 𝑆𝐴𝑅𝐼𝐻,𝐵 hospital in the base governorate, and 𝑃𝑜𝑝𝐵 = source population of the base governorate.

#### 2.3.2 Step 2: Estimation of SARI Hospitalizations Rates in All Governorates

Estimates of SARI hospitalization rates for the other 25 governorates in Egypt were obtained by adjusting Aswan and Damietta governorates rates for the governorate‐specific prevalence of known risk factors for pneumonia and health seeking behavior obtained from the 2015 DHS [8] using a previously described methodology [16, 17, 18, 19, 20]. The prevalence of the risk factors in the two base governorates combined was obtained using the prevalence of each risk factor in each governorate weighted by the population of each governorate (pooled weighted prevalence). Risk factors for pneumonia such as exposure to indoor smoking, air pollution, low birth weight, malnutrition, and nonexclusive breast feeding (the last three included only for children aged < 5 years) were used. Due to the lack of estimates of healthcare seeking behavior for acute respiratory conditions, sexually transmitted infections (STIs) were used as a proxy for differential healthcare seeking between governorates. On sensitivity analysis, we estimated the SARI hospitalization rates without adjustment for STI healthcare seeking. The relative risks of SARI associated with each risk factor were obtained from published literature [21, 22]. An adjustment factor > 1 resulted in a greater SARI hospitalization rate in the given governorate relative to the base governorates and vice versa. The equations used for the adjustments by governorate are provided below.

$${\mathrm{Adj}}_{Y}=\left(1+\sum_{i}\left(P_{i,Y}-P_{i,B}\right)\times \left({\mathrm{RR}}_{i}-1\right)\right),$$

where ${\mathrm{Adj}}_{Y}$ = adjustment factor for governorate *Y* for risk factors of SARI, $P_{i,Y}$ = prevalence of risk factor *i* in governorate *Y*, $P_{i,B}$ = prevalence of risk factor *i* in base governorate, and ${\mathrm{RR}}_{i}$ = relative risk of SARI due to risk factor *i*.

$${\mathrm{RS}}_{H,Y}={\mathrm{RS}}_{H,B}\times {\mathrm{Adj}}_{Y}\times \frac{{\mathrm{DHS}}_{H,Y}}{{\mathrm{DHS}}_{H,B}},$$

where ${\mathrm{RS}}_{H,Y}$ = rate of hospitalized SARI in governorate *Y*, ${\mathrm{DHS}}_{H,Y}$ = proportion of STI cases seeking care in governorate *Y* (from DHS), and ${\mathrm{DHS}}_{H,B}$ = proportion of STI cases seeking care in base governorate (from DHS).

#### 2.3.3 Step 3: Estimation of Influenza‐Associated SARI Hospitalizations Rates in All Governorates

The rates of influenza‐associated SARI hospitalization per governorate were calculated by multiplying the estimated SARI hospitalization rates for each governorate (obtained in Steps 1 and 2) by the influenza positivity rate among SARI cases admitted at all (eight) sentinel hospitals during 2016–2019 using the equation provided below.

$${\mathrm{RI}}_{H,Y}={\mathrm{RS}}_{H,Y}\times \;I,$$

where ${\mathrm{RI}}_{H,Y}$ = rate of hospitalized influenza‐associated SARI in governorate *Y* (including base governorate) and I = proportion of hospitalized SARI cases testing positive for influenza.

#### 2.3.4 Step 4: Estimation of the Numbers of SARI and Influenza‐Associated SARI Hospitalizations in All Governorates

We estimated the number of SARI cases and influenza‐associated SARI hospitalizations per governorate by multiplying the SARI hospitalization rates (obtained in Steps 1 and 2) and influenza‐associated SARI hospitalization rates (obtained in Step 3) by the midyear population in each governorate using the equations provided below. Rates were estimated over the three‐season study period, applied to the corresponding 3‐year population estimates and divided by 3 to obtain mean seasonal numbers of influenza‐associated illness.

$${\mathrm{NS}}_{H,Y}={\mathrm{RS}}_{H,Y}\times P{\mathrm{op}}_{Y},$$

where ${\mathrm{NS}}_{H,Y}$ = number of hospitalized SARI cases in governorate *Y* (including base governorate) and $P{\mathrm{op}}_{Y}$ = population in governorate *Y* (including base governorate).

$${\mathrm{NI}}_{H,Y}={\mathrm{RI}}_{H,Y}\times P{\mathrm{op}}_{Y},$$

where ${\mathrm{NI}}_{H,Y}$ = number of hospitalized influenza‐associated SARI cases in governorate *Y* (including base governorate) and $P{\mathrm{op}}_{Y}$ = population in governorate *Y* (including base governorate).

### 2.4 Estimation of Confidence Interval (CI) for Influenza‐Associated SARI Hospitalizations

For all parameters that were included in the calculations, Monte Carlo simulations over 1000 replications were used to obtain 95% CIs. The sample included (i) the rate of SARI hospitalizations by age, (ii) the prevalence of pneumonia risk factors for each governorate, (iii) the proportion of STI cases seeking care, and (iv) the influenza positivity proportion among SARI cases by age. The lower and upper limits of the 95% CI were the 2.5th and 97.5th percentiles of the estimated values obtained from the 1000 resampled datasets, respectively. The analysis was performed using Stata Version 16 (StataCorp, Texas, USA).

### 2.5 Estimation of the Influenza Disease Burden Across Levels of Severity

The influenza disease burden pyramid tool developed by WHO and the John Hopkins Center for Health Security [11] was used to estimate the national number of influenza‐associated mild/moderate cases, hospitalizations needing critical care, and deaths based on the mean seasonal number of age‐specific influenza‐associated SARI hospitalizations and regions (as obtained in Step 4 above) over the study period. The tool uses a multiplier‐based approach to estimate the influenza‐associated disease burden across levels of severity using available estimates of influenza‐associated hospitalizations or deaths. Rates were obtained by dividing the estimated number of age‐specific influenza‐associated illness across levels of severity by the midyear population at risk for Egypt.

### 2.6 Ethical Approval

The study was deemed nonresearch by the Egyptian Ministry of Health and Population (MoHP) ethics committee and Institutional Review Board (IRB). The study was conducted using surveillance data collected during patients' routine management procedures.

## 3 Results

During the study period, a total of 8188 SARI patients were admitted to all government hospitals of the two base governorates (Damietta and Aswan); of these, 2686 (32.8%) were children aged < 5 years, 4340 (53.0%) were males, and 1418 (17.3%) were admitted to the DCH and AIH sentinel hospitals.

**FIGURE 1:**
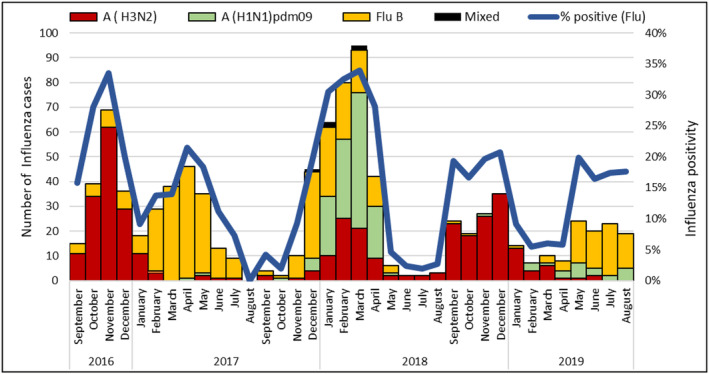
Monthly number of influenza positive specimens and influenza percentage positivity among patients hospitalized with severe acute respiratory infection (SARI) at eight sentinel hospitals in Egypt, September 2016–August 2019. *Note:* This figure illustrates the monthly fluctuations in the number of influenza‐positive specimens and the percentage of influenza positivity among patients hospitalized with SARI at eight sentinel hospitals in Egypt between September 2016 and August 2019. The figure likely shows seasonal patterns of influenza activity and influenza virus types with peaks during the winter months.

A total of 5512 patients with SARI were enrolled and tested for influenza virus at the eight SARI sentinel hospitals situated across Egypt; of these, 932 (17%) tested positive for influenza. The influenza percentage positive was 14.4% (225/1567) among children aged < 5 years and 17.9% (707/3945) among individuals aged ≥ 5 years. Influenza A(H1N1)pdm09, A(H3N2), B, and A/B coinfections accounted for 17.7% (165), 39.0% (364), 42.6% (397), and 0.5% (6) of influenza positive specimens, respectively (Figure 1).

The estimated mean seasonal number of influenza‐associated SARI hospitalizations was 235,957 (95% CI: 204,646–269,353); 85,797 (95% CI: 74,069–98,362; 36.4%) among children aged < 5 years, and 150,160 (95% CI: 130,577–170,991; 63.6%) among individuals aged ≥ 5 years. The estimated SARI hospitalization rate per 100,000 population was 244.3 (95% CI: 211.9–278.9); 364.8 (95% CI: 315–418.3) among children aged < 5 years, and 205.5 (95% CI: 178.7–234) among individuals aged ≥ 5 years (Table 1).

**TABLE 1:** Mean seasonal numbers and rates (per 100,000 population) of severe acute respiratory infections (SARI) hospitalizations and influenza‐associated SARI hospitalizations by age and region in Egypt, September 2016–August 2019.

	SARI hospitalizations				Influenza‐associated SARI hospitalizations			
	Number	95% CIs	Rate	95% CIs	Number	95% CIs	Rate	95% CIs
Age in years (2017 midyear population)								
< 5 (23,516,826)	85,797	74,069–98,362	364.8	315.0–418.3	12,319	10,112–14,642	52.0	43.0–62.0
5–14 (18,776,628)	24,093	19,977–28,828	128.0	106.0–154.0	5288	4107–6677	28.2	21.9–35.6
15–49 (44,707,259)	77,749	68,829–86,802	174.0	154.0–194.0	13,729	11,736–15,863	31.0	26–36
50–64 (7,641,030)	29,620	25,737–33,676	388.0	337.3–441.4	4996	4104–6039	65.4	53.7–79
65+ (1,950,628)	18,698	16,035–21,685	959.0	822.5–1112.7	3109	3109–3999	159.4	121.7–205.0
5+ (73,075,545)	150,160	130,577–170,991	205.5	179.5–234.5	27,122	22,320–32,577	37.1	30.5–44.6
Region (2017 midyear population)								
Urban governorates^a^ (16,231,307)	35,921	31,101–40,994	221.3	191.6–252.6	6065	4983–7295	37.4	30.7–44.9
Upper Egypt^b^ (37,158,038)	96,804	84,162–110,440	261.8	227.6–298.6	16,143	13,299–19,305	43.7	36.0–52.2
Frontier governorates^c^ (1,605,898)	3562	3098–4056	221.8	192.9–252.6	589	490–708	26.7	30.5–44.1
Lower Egypt^d^ (41,777,209)	99,670	86,286–113,863	238.6	206.5–272.5	16,644	13,660–19,911	39.8	32.7–47.7
Total (96,592,371)	235,957	204,646–269,353	244.3	211.9–278.9	39,445	32,431–47,219	40.8	33.6–48.9

Abbreviation: CIs: confidence intervals.

^a^ Urban governorates: Alexandria, Port Said, Cairo, and Suez.

^b^ Upper Egypt: Aswan, Assuit, Beni Suef, Fayoum, Giza, Luxor, Minya, Qena, and Sohag.

^c^ Frontier governorates: Matrouh, New Valley, North Sinai, Red Sea, and South Sinai.

^d^ Lower Egypt: Beheira, Dakahlia, Damietta, Gharbia, Ismailia, Kafr el‐Sheikh, Menofia, Qualyubia, and Sharqia.

**FIGURE 2:**
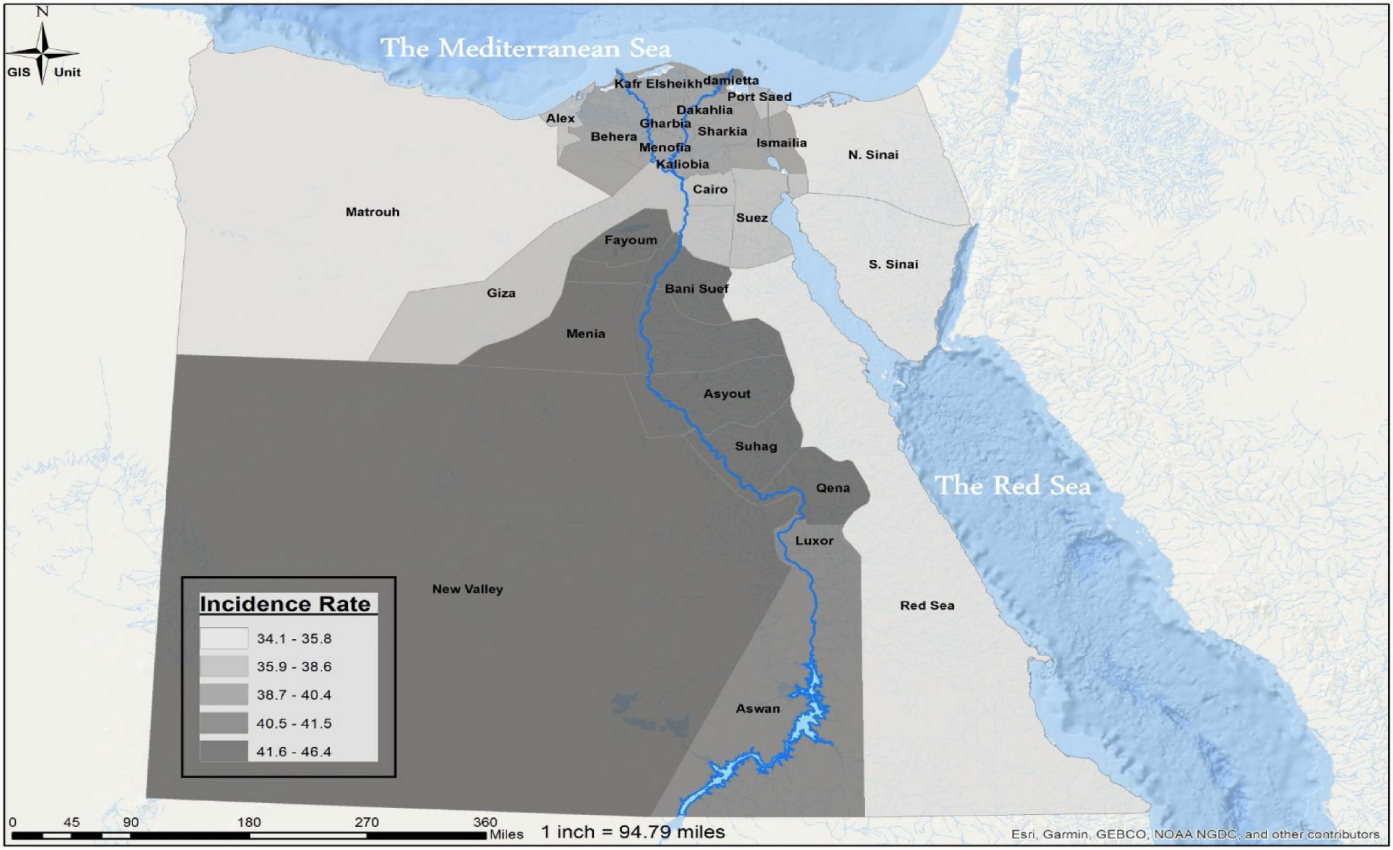
Mean seasonal rate (per 100,000 population) of influenza‐associated severe acute respiratory infection hospitalization by Governorate, Egypt, September 2016–August 2019. *Note:* This map visualizes the geographical distribution of influenza‐associated severe acute respiratory infection (SARI) hospitalization rates across different governorates in Egypt between September 2016 and August 2019. The map uses color‐coding to represent the mean seasonal rate of SARI hospitalizations per 100,000 population in each governorate. Darker shades indicate higher rates of hospitalization, while lighter shades represent lower rates.

The estimated mean seasonal number of influenza‐associated SARI hospitalizations was 39,445 (95% CI: 32,431–47,219); 12,319 (95% CI: 10,112–14,642; 41.6%) among children aged < 5 years, and 27,122 (95% CI: 22,320–32,577; 58.4%) among individuals aged ≥ 5 years. The estimated rate of influenza‐associated SARI hospitalizations per 100,000 population was 40.8 (95% CI: 33.6–48.9); 52.0 (95% CI: 43.0–62.3) among children aged < 5 years, and 37.1 (95% CI: 30.5–44.6) among individuals aged ≥ 5 years (Tables 1). The highest influenza‐associated SARI hospitalization rate (159.4; 95% CI: 121.7–205.0 per 100,000 population) was among individuals aged ≥ 65 years and in the Upper Egypt region (43.7; 95% CI: 36–52.2 per 100,000 population) (Table 1). The mean seasonal rate of influenza‐associated SARI hospitalization over the study period by Governorate is provided in Figure 2. On sensitivity analysis without adjusting for STI healthcare seeking, the estimated numbers of influenza‐associated SARI were similar (40,275; 95% CI: 33,310–47,806) to STI healthcare seeking adjusted numbers.

The estimated mean seasonal number of influenza‐associated mild/moderate cases, hospitalized noncritically and critically ill patients, and deaths was 16,425,938 (95% CI: 1,150,888–40,409,614), 30,335 (95% CI: 9971–670,288), 9110 (95% CI: 580–16,321), and 2660 (95% CI: 154–9908), respectively. The highest rate of influenza‐associated mild/moderate cases (22,932; 95% CI: 825–25,546.3) was among children aged 5–14 year, whereas the highest rate of influenza‐associated critically ill patients (58.6; 95% CI: 0.6–111.7 per 100,000 population) and deaths (56.0; 95% CI: 0.6–111.0 per 100,000 population) was among individuals aged > 65 years (Table 2).

**TABLE 2:** Estimated average seasonal numbers and rates (per 100,000 population) of influenza‐associated illness by age, region, and level of severity in Egypt, September 2016–August 2019.

	Influenza‐associated deaths				Influenza‐associated SARI hospitalizations (critically ill)			
	Number	95% CIs	Rate	95% CIs	Number	95% CIs	Rate	95% CIs
Age in years								
< 5	118	48–3095	0.5	0.2–13.0	1849	181–5098	7.9	0.3–20.0
5–14	151	21–1328	0.8	0.1–7.0	941	78–2188	5.2	0.6–39.0
15–49	909	54–3449	2.1	0.1–8.0	3487	202–5681	7.9	2.0–136.0
50–64	386	19–1255	5.0	0.3–16.0	1679	73–2067	21.9	7.0–439.0
65+	1087	12–2023	56.0	0.6–111.0	1144	46–2287	58.6	0.6–111.7
5+	2533	106–6813	3.0	0.1–9.0	7251	399–11,223	10.1	0.4–13.0
Region								
Urban governorates^a^	469	24–1524	2.9	0.2–9.0	1485	89–2510	9.1	0.4–24.0
Upper Egypt^b^	1041	63–4056	2.9	0.1–7.0	3648	237–6681	10.0	0.4–25.0
Frontier governorates^c^	36	2–148	2.3	0.3–16.0	132	9–244	8.3	0.5–33.0
Lower Egypt^d^	1114	65–4181	2.7	0.1–7.0	3845	245–6888	9.4	0.4–23.0
Total	2660	154–9908	2.8	0.1–7.0	9110	580–16,321	9.6	0.4–24.4

	Influenza‐associated SARI hospitalizations (not critically ill)				Influenza‐associated mild/moderate cases			
	Number	95% CIs	Rate	95% CIs	Number	95% CIs	Rate	95% CIs
Age (in years)								
< 5	10,470	289–25,464	44.3	1.7–110.0	1,809,908	359,468–4,376,694	7749.2	1531.3–18,741.5
5–14	4347	523–36,921	23.3	2.4–177.0	4,300,519	154,304–8,878,721	22,932	825.7–25,546.3
15–49	10,242	3416–229,750	23.7	6.0–399.0	9,801,683	400,612–19,877,639	22,134.3	5874.0–45,243.0
50–64	3317	1463–97,907	44.3	13.0–865.0	485,712	145,783–1,774,979	6422.6	1928.0–24,538.1
65+	1965	832.4–3901.5	100.7	51.5–203.2	27,142	13,846.3–55,783.9	1551.4	758.5–3123.8
5+	19,871	2686–189,502	27.0	3.0–68.0	14,615,056	791,420–28,635,904	20,037.7	1097.0–2237.60
Region								
Urban governorates^a^	4580	1772–118,961	28.2	11.6–724.0	2,535,562	176,977–5,154,773	15,621.4	591.0–38,430.0
Upper Egypt^b^	12,497	3906–262,938	34.4	1.0–85.0	6,694,670	471,111–15,735,996	18,442.1	697.0–45,370.0
Frontier governorates^c^	457	134–9080	28.8	1.0–82.0	254,352	17,187–723,260	1602.7	606.0–39,413.0
Lower Egypt^d^	12,801	4189–281,638	31.2	1.0–77.0	6,941,354	485,730–15,913,991	16,896.1	639.0–41,567.0
Total	30,335	9971–670,288	31.9	1.0–79.0	16,425,938	1,150,888–40,409,614	17,253.7	652.0–42,446.0

Abbreviation: CIs: confidence intervals.

^a^ Urban governorates: Alexandria, Port Said, Cairo, and Suez.

^b^ Upper Egypt: Aswan, Assuit, Beni Suef, Fayoum, Giza, Luxor, Minya, Qena, and Sohag.

^c^ Frontier governorates: Matrouh, New Valley, North Sinai, Red Sea, and South Sinai.

^d^ Lower Egypt: Beheira, Dakahlia, Damietta, Gharbia, Ismailia, Kafr el‐Sheikh, Menofia, Qualyubia, and Sharqia.

## 4 Discussion

We estimated the burden of influenza‐associated illness in Egypt over three influenza seasons. The results indicate a sizable number of influenza‐associated illness across levels of severity, age groups, and regions.

During 2016–2019, we estimated that, on average, approximately 17.3% (rate: 17,253 per 100,000 population) of the Egyptian population was affected by influenza‐associated mild/moderate illness seasonally. Whereas burden estimates of influenza‐associated mild/moderate illness in the WHO Eastern Mediterranean Region and Sub‐Saharan Africa are scarce, our estimated rates (per 100,000 population) are generally consistent with that from studies conducted in Lebanon (16,420) [23] and South Africa (19,550) [24]. Similar to other studies [23, 24], we found higher rates of influenza‐associated mild/moderate illness among school‐aged children and young adults compared to other age groups, highlighting the importance of these individuals in influenza virus transmission [23, 24].

Our estimated rate (per 100,000 population) of influenza‐associated SARI hospitalization (40.8) is similar to that of a previous study conducted in Damanhur district, Lower Egypt (44.1) [7], and is generally consistent with estimates from countries in the WHO Eastern Mediterranean Region and Sub‐Saharan Africa: 15.9–61.3 in Iran [25], 48.1 in Lebanon [23], 20.6 in Oman [26], 48.5 in the Democratic Republic of Congo [16], 33.0 in Ghana [27], 20.8 in Kenya [17], 30.0 in Madagascar [19], 34.7 in Rwanda [28], 46.7 in South Africa [24], 34.0 in Uganda [29], and 43.9 in Zambia [18]. In our study, we found the highest rate of influenza‐associated SARI hospitalization among individuals aged ≥ 65 years followed by children aged < 5 years. This pattern is similar to that observed in other Eastern Mediterranean countries [23, 26], whereas in most of sub‐Saharan countries, the highest rates of influenza‐associated SARI hospitalization were found among children aged < 5 years followed by individuals aged ≥ 65 years [16, 17, 18, 19, 24, 27, 28, 29]. These differences in hospitalization rates may be attributed to different population age structure and differential healthcare seeking behavior and prevalence of risk factors for severe influenza across different age strata in different countries.

Whereas burden estimates of influenza‐associated respiratory deaths in the WHO Eastern Mediterranean Region and Sub‐Saharan Africa are scarce, our estimated rates (2.8 per 100,000 population) are generally consistent with that from studies conducted in Lebanon (5.8 per 100,000 population) [23], Oman (0.9 per 100,000 population) [26], Kenya (10.5 per 100,000 population) [30], and South Africa (3.5 per 100,000 population) [24] as well as estimates for the WHO East Mediterranean Region (2.1–13.4 per 100,000 population) from a global study [31]. Similar to other studies [23, 24, 26, 30, 31], we found the highest rates of influenza‐associated deaths among individuals aged ≥ 65 years.

The Egyptian MoHP made influenza vaccine available commercially every year and emphasized the importance of seasonal influenza vaccination as a preventive measure against severe outcome particularly for those who are most at risk of complications related to influenza [32]. Vaccination against influenza is recommended in Egypt for patients with chronic illnesses, such as diabetes and kidney disease, immunologic disorders, HIV/AIDS, and morbid obesity, and healthcare workers (HCWs), veterinary services workforce, airline personnel, and pilgrims [33]. Although in Egypt influenza vaccination is strongly recommended for HCWs to protect them and reduce the risk of disease transmission in healthcare settings, vaccination coverage among HCWs remains suboptimal (31%), while no national data are available on influenza vaccination coverage among the elderly or other target groups [34, 35]. Estimating vaccination coverage in older people and other target groups is essential as national vaccination policies are implemented based on coverage rates to enhance vaccine uptake and thus reduce the burden of influenza. There needs to be more effort put into increasing influenza vaccine demand, uptake, and acceptance together with enhanced immunization strategies (e.g., high‐dose or adjuvant formula) to be largely implemented in the elderly to reduce influenza‐associated hospitalization and mortality [36].

## 5 Limitations

Our study has limitations that warrant discussion. First, we used the STI healthcare‐seeking behavior as a proxy for ARI healthcare‐seeking behavior in Egypt due to the unavailability of that for ARI. Second, our hospitalization estimates focus on patients hospitalized with SARI. In this regard, we may not have included patients who did not seek healthcare or that did not present with a clinical presentation compatible with the SARI case definition; therefore, our estimates should be viewed as minimum estimates. Third, in our estimation approach, we used data from governmental hospitals, which include the majority of ARI hospitalizations but did not include private hospitals. Fourth, the national influenza surveillance system is conducted at MoHP hospitals; other healthcare facilities in private and other governmental healthcare facilities such as university and university hospitals were not included. Last, we did not apply weekly or monthly influenza proportion positive (as suggested in the WHO manual) to national estimates of SARI rates as national extrapolation was not implemented weekly or monthly and because the number of weekly or monthly samples tested for influenza virus detection within each age strata was too small, resulting in reduced accuracy. Similarly, the annual number of samples tested for influenza virus detection in certain age strata (especially in older age groups) were small, hindering the possibility of obtaining accurate seasonal estimates.

## 6 Conclusion

The WHO manual and tools proved effective in estimating the national influenza disease burden in Egypt. Countries with robust influenza sentinel surveillance could benefit from WHO methods to estimate incidence rates from selected sentinel sites and extrapolating nationally. A higher burden of severe influenza (i.e., hospitalizations, critical cases, and deaths) was found among individuals aged ≥ 65 followed by children aged < 5 years. The reduction of influenza‐related morbidity and mortality in Egypt requires improving influenza vaccination coverage, educating the public about the importance of influenza vaccination, maintaining surveillance on ARI, and monitoring influenza burden to detect increases in morbidity and mortality. The results of this study could guide preventive strategies to reduce influenza disease burden, especially among young children and the elderly. Further studies are required to assess: (i) the burden of influenza disease during the post‐COVID‐19 pandemic period; (ii) influenza vaccine coverage among high‐risk groups; and (iii) their knowledge, attitude, and practice regarding influenza vaccination.

## Author Contributions

**Manal Fahim:** investigation, supervision. **Ola Deghedy:** data curation, investigation, formal analysis, writing – original draft. **Walaa Alim:** investigation, writing – original draft, formal analysis, data curation. **Reham Kamel:** data curation. **Hossam Hassan:** writing – review and editing. **Amira Mohsen:** funding acquisition. **Rania Attia:** writing – review and editing. **Hala Abou El Naja:** writing – review and editing. **Salma Afifi:** writing – review and editing. **Stefano Tempia:** methodology, validation, formal analysis. **Amr Kandeel:** conceptualization.

## Disclosure

The findings and conclusions in this report are those of the authors and do not necessarily represent the official position of the World Health Organization or the Egypt MoHP.

## Ethics Statement

The study was deemed nonresearch by the Egyptian Ministry of Health and Population (MoHP) ethics committee and Institutional Review Board (IRB). The study was conducted using surveillance data collected during patients' routine management procedures.

## Conflicts of Interest

The authors declare no conflicts of interest.

### Peer Review

The peer review history for this article is available at https://www.webofscience.com/api/gateway/wos/peer‐review/10.1111/irv.70061.

## Data Availability

The datasets generated and/or analyzed during the current study are not publicly available due to data privacy restrictions but are available from Egypt Ministry of Health and Population on reasonable request.
